# Collapsing glomerulopathy is likely a major contributing factor for worse allograft survival in patients receiving kidney transplants from black donors

**DOI:** 10.3389/fmed.2024.1369225

**Published:** 2024-03-14

**Authors:** Lanny T. DiFranza, Emily Daniel, Geo Serban, Steven M. Thomas, Dominick Santoriello, Lloyd E. Ratner, Vivette D. D’Agati, Elena-Rodica Vasilescu, Syed Ali Husain, Ibrahim Batal

**Affiliations:** ^1^Pathology and Cell Biology, Columbia University Irving Medical Center, New York, NY, United States; ^2^Medicine, Nephrology, Columbia University Irving Medical Center, New York, NY, United States; ^3^Surgery, Columbia University Irving Medical Center, New York, NY, United States

**Keywords:** kidney transplantation, kidney pathology, collapsing glomerulopathy, racial disparities, allograft outcomes

## Abstract

Although a few registry-based studies have shown associations between receiving kidney allografts from Black donors and shorter allograft survival, detailed, large, single-center studies accounting for common confounding factors are lacking. Furthermore, pathologic alterations underlying this potential disparity have not been systematically studied. We performed a retrospective clinical-pathological study of kidney transplant recipients who received kidney allografts from either Black (*n* = 407) or White (*n* = 1,494) donors at Columbia University Irving Medical Center from 2005 to 2018, with median follow-up of 4.5 years post-transplantation. Black donor race was independently associated with allograft failure (adjusted HR = 1.34, *p =* 0.02) and recipients of kidney allografts from Black donors had a higher incidence of collapsing glomerulopathy [7.4% vs. 1.9%, OR = 4.17, *p <* 0.001]. When causes of allograft failure were examined, only allograft failure following development of collapsing glomerulopathy was more frequent in recipients of allografts from Black donors [15% vs. 5%, OR = 3.16, *p* = 0.004]. Notably, when patients who developed collapsing glomerulopathy were excluded from analysis, receiving kidney allografts from Black donors was not independently associated with allograft failure (adjusted HR = 1.24, *p* = 0.10). These findings revealed that, compared with recipients of kidney allografts from White donors, recipients of kidneys from Black donors have modestly shorter allograft survival and a higher probability of developing collapsing glomerulopathy, which negatively impacts allograft outcome. Identification of collapsing glomerulopathy risk factors may help decrease this complication and improve allograft survival, which optimally may reduce racial disparities post-transplantation.

## Introduction

While kidney transplantation is the optimal treatment for kidney failure, apparent racial and ethnic disparities persist after transplantation. Several registry-based studies have shown that kidneys from deceased Black donors exhibit shorter allograft survival compared to kidneys transplanted from White donors (1, 2). This has led to the inclusion of Black donor race in the calculation of the kidney donor profile index (KDPI) used to evaluate deceased donor kidney quality in the United States, and, thus, has contributed to an increased likelihood of kidney non-procurement and non-utilization of organs from deceased Black donors (3).

Post-transplant collapsing glomerulopathy (CG) is an infrequent complication of the kidney allograft that has detrimental effects on allograft survival (4). Whereas a few studies have suggested an association between CG and receiving kidney allografts from Black donors, especially those harboring Apolipoprotein L1 gene (*APOL1*) high-risk genotypes (4–6), systematic comparisons of the incidence and prognosis of post-transplant CG between different donor races/ethnicities are currently lacking.

We hypothesized that the association between Black donor race and inferior allograft survival is mediated by the development of CG in a minority of transplants. To address this issue, we performed the largest single-center clinical-pathologic study to date in which we aimed to confirm the disparity in outcomes between recipients of kidney allografts from Black vs. White donors, after adjusting for major confounding factors, and to investigate the histopathologic changes that may explain the observed disparities.

## Materials and methods

With approval of the Institutional Review Board at Columbia University, we conducted a retrospective study of kidney transplant patients who received kidney allografts from self-identified Black and White donors at the Columbia University Irving Medical Center (CUIMC) between January 1, 2005, and December 31, 2018. Briefly, all patients who underwent kidney transplantation at CUIMC were identified, and only patients who received allografts from donors identified as Black or non-Hispanic White were included.

Forty-six patients received more than one kidney allograft (including 41 recipients of kidneys from White donors and 5 recipients of kidneys from Black donors). For statistical purposes, these subjects were included once for each corresponding transplant. Our final cohort included 1901 allografts, combining 407 transplants from Black donors and 1,494 from White donors.

In our center, the majority of patients are maintained on tacrolimus and mycophenolate sodium, without maintenance corticosteroids. Clinical parameters were extracted from medical records. These included recipient and donor demographics (age, sex, and race), cause of native kidney failure, history of previous kidney transplant, induction immunosuppression therapy, donor-recipient human leukocyte antigen (HLA) mismatch (0–6 based on A, B, and DR antigens), and the presence of pre-transplant circulating donor-specific antibodies [DSA, defined as mean fluorescence intensity >1,000 as assessed by Luminex single-antigen bead assay (One Lambda Inc., Canoga Park, CA)]. To provide a measurable proxy of patients’ socioeconomic status, median household income according to the ZIP code of each recipient’s residence was used (7). We used the median ZIP code household income data from the American Community Survey 2014 5-Year Estimates Subject Tables, which included the 5-year interval (2010–2014) covering the middle of the study period.

### Follow-up

**Figure 1:**
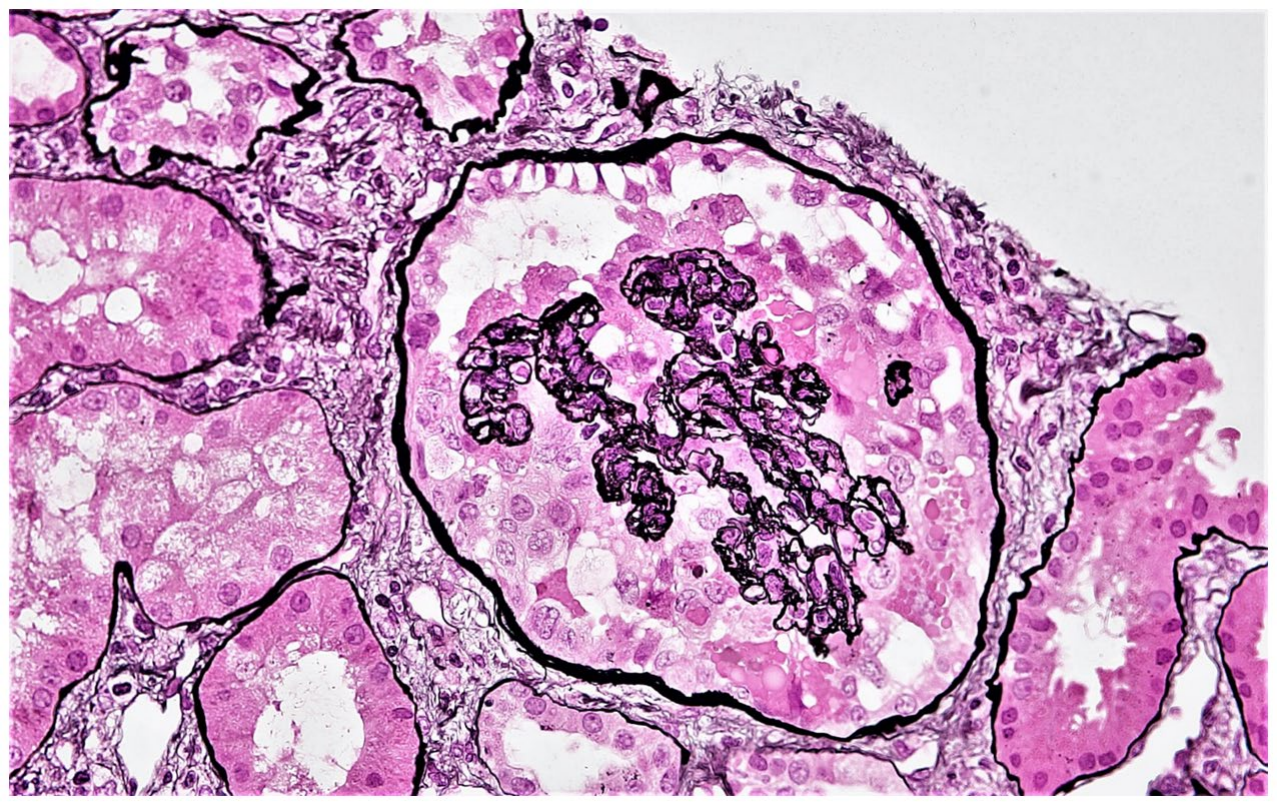
Representative photomicrograph showing collapsing glomerulopathy in a kidney allograft. A glomerulus displaying features of collapsing glomerulopathy in a kidney allograft biopsy. This glomerulus displays global collapse of the glomerular tuft, wrinkled and retracted glomerular basement membranes, and hypertrophy and hyperplasia of the overlying glomerular epithelial cells (Jones methenamine silver stain). Original magnification ×400.

Patients were censored at the end of the follow-up period (December 31, 2019). Death-censored allograft failure (determined as re-initiation of maintenance kidney replacement therapy or re-transplantation) was considered the primary outcome, while the development of CG (Figure 1) was considered a secondary outcome. As explained previously (4), CG was defined using the Columbia classification of focal segmental glomerulosclerosis (FSGS) as ≥1 glomerulus with segmental or global wrinkling and retraction of the glomerular basement membranes, accompanied by hypertrophy and hyperplasia of overlying glomerular epithelial cells (8), and the diagnosis was confirmed by two pathologists.

In patients who did not develop CG, we aimed to assess progression of other histologic changes over time, depending on the findings extracted from pathology reports of the post-reperfusion biopsy and last available allograft biopsy performed beyond 1 year after transplantation and before the end of the follow-up period. At CUIMC, post-reperfusion biopsies are routinely performed, while subsequent kidney allograft biopsies are performed for clinical indications (elevation of serum creatinine or proteinuria), or per-protocol, mainly in patients with pre-transplant DSA or a positive pre-transplant flow cytometry crossmatch (at 1 week, 2 weeks, 1 month, 3 months, 6 months, 1 year, 2 years, 3 years, and 5 years after transplantation). All biopsies were processed for light microscopy using the standard procedure employed in processing of kidney biopsy specimens, including staining with hematoxylin and eosin, periodic acid-Schiff, Masson trichrome, and Jones methenamine silver.

Assessed histologic parameters included the number of glomeruli present, number and percentage of glomeruli displaying each of global glomerulosclerosis (GGS) and non-collapsing FSGS, percentage of interstitial fibrosis/tubular atrophy (IFTA), and histologic scores for arteriosclerosis (0–3) and arteriolar hyalinosis (0–3), as assessed using Banff criteria (9).

The rate of progression of IFTA over time in the same subjects was calculated using the following equation: [ΔIFTA = (%IFTA in last allograft biopsy – %IFTA in post-reperfusion biopsy) / number of months post-transplantation between these two biopsies]. Analogous equations were used to determine the rates of progression of GGS, FSGS, arteriosclerosis, and arteriolar hyalinosis over time, substituting the % of IFTA with each of % of GGS, % of FSGS, arteriosclerosis score, and arteriolar hyalinosis score, respectively.

If any required data were missing from the pathology reports, a review of the original biopsy slides was undertaken by two pathologists (LDF and IB) to obtain the missing data. If a specific histologic parameter was unable to be evaluated due to sampling limitations, the corresponding data point was labeled as missing.

### Statistical analysis

Statistical analysis was performed using Prism version 9 (GraphPad Inc., San Diego, CA) and SPSS Statistics version 27 (IBM, Armonk, NY). Continuous data were presented as median and interquartile range (IQR; 25th and 75th percentile), and compared using the Mann–Whitney test, while categorical variables were compared using Fisher’s exact test. Kaplan–Meier methodology and log-rank test were utilized to assess allograft survival, while Cox proportional hazards models were constructed to account for confounders. Variables with *p* < 0.10 on univariate analyses and variables that were different between patients receiving kidney allografts from Black vs. White donors were included in the multivariable analyses. Individuals with missing information for a tested predictor were excluded from the corresponding univariable time-to-event analysis, and those with missing data in one or more predictors in the multivariable analyses were also excluded from the latter analyses. *p* values <0.05 with two-sided hypothesis testing were considered statistically significant.

## Results

### Demographic, clinical, and pathologic features

Between January 2005 and December 2018, 1901 kidney allografts were transplanted from either self-identified Black (*n* = 407, 21%) or White (*n* = 1,494, 79%) donors at CUIMC. Overall, kidney allograft recipients had a median age of 53 years, 38% were female, 22% self-identified as Black, 15% had undergone prior kidney transplantation, and 52% received kidney allografts from deceased donors (Table 1). The donors had an average age at donation of 46 years and 49% were female. The median number of HLA mismatches was 4, and 19% of the kidney allograft recipients had pre-transplant DSA.

**Table 1:** Demographics and clinical characteristics of recipients of kidney allografts from Black vs. White donors.

	Total (*n* = 1901)	Recipients of kidneys from Black donors (*n* = 407)	Recipients of kidneys from White donors (*n* = 1,494)	*p* value (Black vs. White donors)
Recipient age (median, years)	53 (41, 62)	51 (39, 61)	54 (42, 62)	**0.03**
Recipient female sex	728/1901 (38%)	180/407 (44%)	548/1494 (37%)	**0.006**
Recipient Black race	418/1901 (22%)	215/407 (53%)	203/1494 (14%)	**<0.001**
Donor age^1^ (median, years)	46 (34, 54)	42 (29, 52)	46 (35, 55)	**<0.001**
Donor female sex	937/1901 (49%)	199/407 (49%)	738/1494 (49%)	0.87
Deceased donor	994/1901 (52%)	250/407 (61%)	744/1494 (50%)	**0.001**
# of HLA mismatches^2^0 Mismatch 1 Mismatch 2 Mismatch 3 Mismatch 4 Mismatch 5 Mismatch 6 Mismatch	4 (3, 5) 117/1900 (6%) 42/1900 (2%) 173/1900 (9%) 312/1900 (16%) 429/1900 (23%) 552/1900 (29%) 275/1900 (15%)	4 (3, 5) 11/407 (3%) 7/407 (2%) 30/407 (7%) 71/407 (18%) 87/407 (21%) 130/407 (32%) 71/407 (17%)	4 (3, 5) 106/1493 (7%) 35/1493 (2%) 143/1493 (10%) 241/1493 (16%) 342/1493 (23%) 422/1493 (28%) 204/1493 (14%)	**0.001** **<0.001** 0.57 0.21 0.57 0.55 0.16 0.06
Pre-transplant DSA^3^	353/1900 (19%)	86/407 (21%)	267/1493 (18%)	0.15
Etiology of native kidney failure				
Diabetes mellitus Glomerulonephritis Hypertension Cystic changes FSGS Obstruction/reflux Others Unknown	459/1901 (24%) 382/1901 (20%) 323/1901 (17%) 193/1901 (10%) 156/1901 (8%) 54/1901 (3%) 299/1901 (16%) 35/1901 (2%)	102/407 (25%) 75/407 (18%) 79/407 (19%) 26/407 (6%) 56/407 (14%) 12/407 (3%) 51/407 (13%) 6/407 (2%)	357/1494 (24%) 307/1494 (21%) 244/1494 (16%) 167/1494 (11%) 100/1494 (7%) 42/1494 (3%) 248/1494 (16%) 29/1494 (2%)	0.65 0.36 0.16 **0.004** **<0.001** 0.87 0.05 0.68
Previous Transplantation^4^	292/1899 (15%)	59/407 (15%)	233/1492 (16%)	0.64
Induction therapy				
Depletion therapy Thymoglobulin Alemtuzumab Non-depletion IL2R Inhibitor No induction	1567/1901 (82%) 1286/1901 (67%) 281/1901 (15%) 333/1901 (18%) 323/1901 (17%) 11/1901 (1%)	338/407 (83%) 279/407 (69%) 59/407 (14%) 69/407 (17%) 66/407 (16%) 3/407 (1%)	1229/1494 (82%) 1007/1494 (67%) 222/1494 (15%) 264/1494 (18%) 257/1494 (17%) 8/1494 (1%)	0.77 0.68 0.94 0.77 0.71 0.71
Median ZIP code household income^5^	71,494 (4,7,050, 9,6,454)	59,653 (42,587, 88,293)	74,784 (51,961, 98,532)	**<0.001**

DSA, donor-specific antibodies; FSGS, focal segmental glomerulosclerosis; HLA, human leukocyte antigen; IL2R, interleukin-2 receptor.

1 Donor age: data on donor age were unavailable for 6 kidney donors (including 3 Black donors and 3 White donors).

2 HLA-mismatch: data on number of HLA mismatches were unavailable for 1 kidney donor-recipient pair (with a White donor).

3 Pre-transplant DSA: data regarding presence of pre-transplant DSA were unavailable for 1 kidney donor-recipient pair (from a White donor).

4 Previous transplantation: data regarding previous renal transplantation were unavailable for 2 kidney recipients (both receiving kidneys from White donors).

5 Median ZIP code household income: data regarding household income were unavailable for 21 kidney recipients (including 4 Black donors and 17 White donors).

The most commonly reported etiology of native kidney failure among allograft recipients was diabetes mellitus (24%), followed by glomerulonephritis (20%), and hypertension (17%). The majority of recipients (82%) received depleting induction therapy, including 67% who received thymoglobulin (Table 1).

Recipients of kidney allografts from Black donors (*n* = 407) were more likely to be Black than those receiving allografts from White donors (53% vs. 14%, *p* < 0.001; Table 1). Additionally, recipients of kidney allografts from Black donors were more likely to be younger (median age: 51 vs. 54 years, *p* = 0.03) and female (44% vs. 37%, *p* = 0.006), and to have lower household income (*p* < 0.001). Recipients of kidney allografts from Black donors were also more likely to have developed native kidney failure attributed to FSGS (14% vs. 7%, *p* < 0.001) and to receive kidneys from deceased donors (61% vs. 50%, *p* = 0.001) that were younger (median age: 42 vs. 46, *p* < 0.001), and HLA-mismatched (97% vs. 93%, *p* < 0.001; Table 1).

### Follow-up

**Figure 2:**
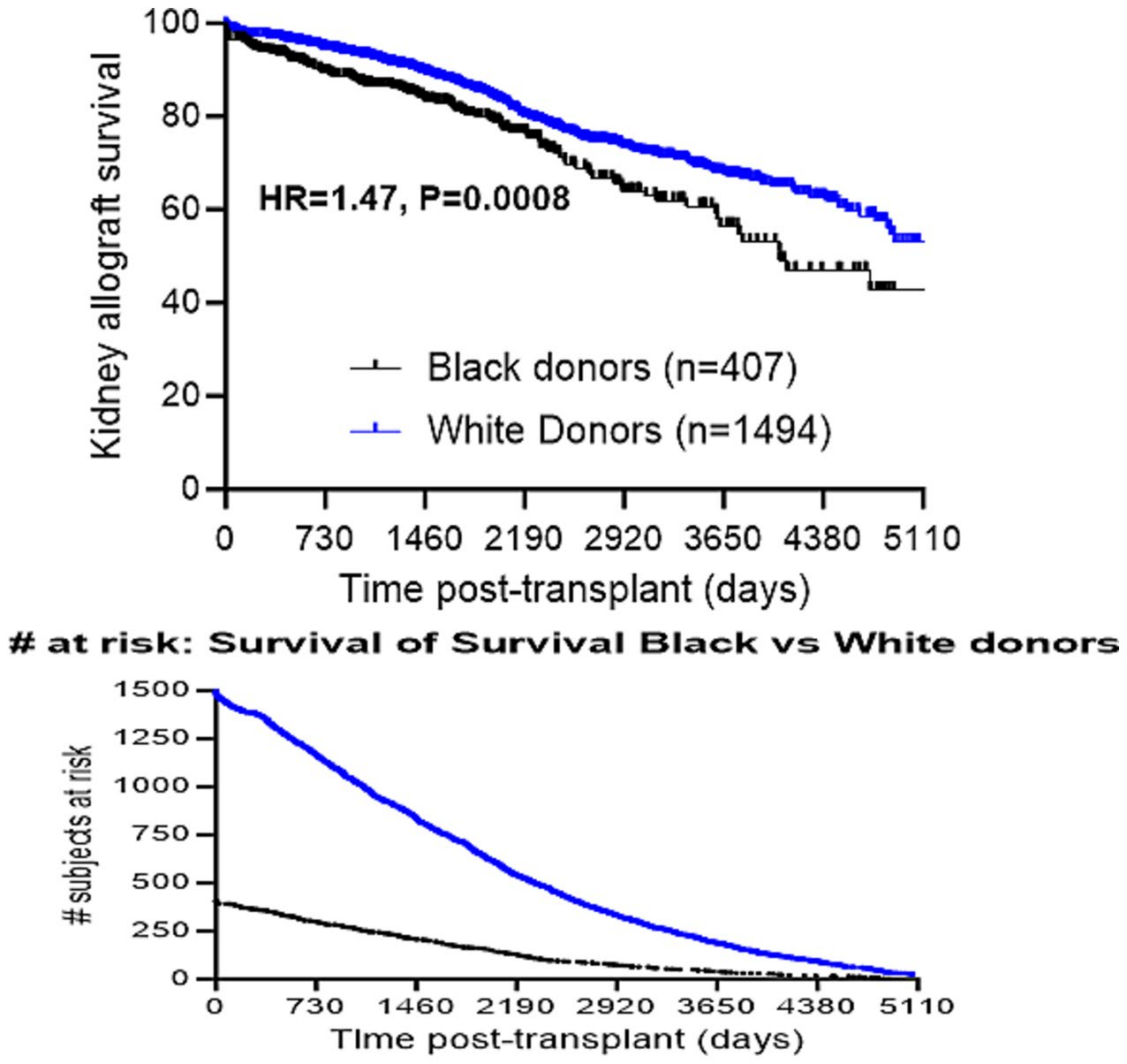
Comparison of allograft survival in recipients of kidney allografts from Black vs. White donors.

Kidney allograft recipients were followed for a median of 4.5 years post-transplantation (IQRs: 2.2, 7.3 years). Of these, 388 (20%) experienced graft failure during follow-up. Notably, allograft survival was shorter for recipients of Black donor kidneys than for recipients of White donor kidneys (HR = 1.47, *p* = 0.0008; Figure 2). To study the independent effect of receiving a kidney allograft from a Black donor on allograft survival, we performed a multivariate analysis that included all variables with *p* < 0.1 on univariate analyses, in addition to variables that were different between patients who received kidney allografts from Black vs. White donors. The latter analysis revealed that receiving a kidney from a Black donor was still associated with a significant, albeit modest, increased risk of allograft failure (aHR = 1.34, *p* = 0.02; Table 2). Other independent predictors included receiving a kidney from a deceased donor (aHR = 2.01, *p* < 0.001), the presence of pre-transplant DSA (aHR =1.42, *p* = 0.008), and receiving a kidney from an older donor (aHR = 1.01 per year, *p* = 0.03; Table 2).

**Table 2:** Univariable and multivariable analyses of death-censored allograft survival.

Variables	Univariable (*n* = 1901)			Multivariable (*n* = 1872), N events = 383	
	N events	HR (95% CI)	*p* value	aHR (95% CI)	*p* value
Recipient age at transplant (per each year)	388	1.00 (0.99–1.00)	0.49	0.99 (0.99–1.00)	0.12
Recipient female gender	388	0.94 (0.77–1.16)	0.58	0.88 (0.71–1.08)	0.22
Recipient Black race	388	1.38 (1.11–1.73)	**0.004**	1.08 (0.85–1.38)	0.54
Donor age at transplant (per each year)^1^	387	1.00 (0.99–1.01)	0.66	1.01 (1.00–1.02)	**0.03**
Donor female sex	388	0.99 (0.81–1.21)	0.92		
Donor Black race	388	1.47 (1.17–1.85)	**0.001**	1.34 (1.05–1.71)	**0.02**
Allograft from Deceased donor	388	2.10 (1.69–2.60)	**<0.001**	2.01 (1.57–2.57)	**<0.001**
Previous transplant^2^	388	1.47 (1.16–1.88)	**0.002**	1.28 (0.97–1.68)	0.08
Pre-transplant DSA^3^	388	1.62 (1.29–2.04)	**<0.001**	1.42 (1.10–1.84)	**0.008**
# HLA mismatches (per antigen: 0–6)^4^	388	1.12 (1.05–1.20)	**0.001**	1.03 (0.96–1.11)	0.46
Induction with Depleting therapy	388	1.18 (0.90–1.54)	0.23		
Native kidney failure due to DM	388	1.05 (0.83–1.32)	0.71		
Native kidney failure due to FSGS	388	1.47 (1.07–2.02)	**0.02**	1.29 (0.93–1.80)	0.13
Median ZIP code household income (per each $10,000/year)^5^	383	0.96 (0.93–0.99)	**0.007**	1.00 (0.96–1.03)	0.84

DM, diabetes mellitus; DSA, donor-specific antibody; FSGS, focal segmental glomerulosclerosis.

1 Donor age: data on donor age were unavailable for 6 kidney donors (including 3 Black donors and 3 White donors).

2 Previous transplantation: data regarding previous renal transplantation were unavailable for 2 kidney recipients (both receiving kidneys from White donors).

3 Pre-transplant DSA: data regarding presence of pre-transplant DSA were unavailable for 1 kidney recipient (from White donor).

4 HLA-mismatch: data on number of HLA mismatches were unavailable for 1 kidney donor-recipient pair (with a White donor).

5 Median ZIP code household income: data regarding household income were unavailable for 21 kidney recipients (including 4 Black donors and 17 White donors).

**Figure 3:**
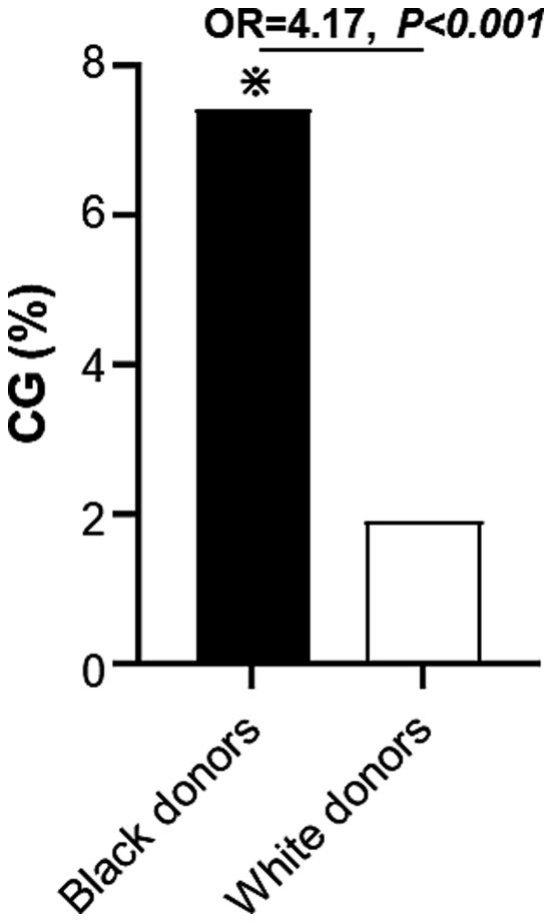
Incidence of collapsing glomerulopathy during follow-up period in recipients of kidney allografts from Black vs. White donors. CG, collapsing glomerulopathy.

When the secondary outcome of CG was assessed, CG was more frequent in kidney allografts procured from Black donors [30/407 (7.4%) recipients of allografts from Black donors vs. 28/1494 (1.9%) recipients of allografts from White donors, OR = 4.17, *p* < 0.001; Figure 3]. This increased frequency of CG in kidneys from Black donors was observed in recipients of kidneys from both living donors [9/157 (6%) Black donors vs. 9/750 (1%) White donors, OR = 5.0, *p* = 0.001] and deceased donors [21/250 (8%) Black donors vs. 19/744 (3%) White donors, OR = 3.5, *p* < 0.001]. When the potential relationship between Black recipient race and CG was examined among patients who received kidneys from Black donors, no difference in the frequency of Black recipients was found between those who developed CG [15/30 (50%)] and those who did not [200/377 (53%), *p = 0.85*].

**Figure 4:**
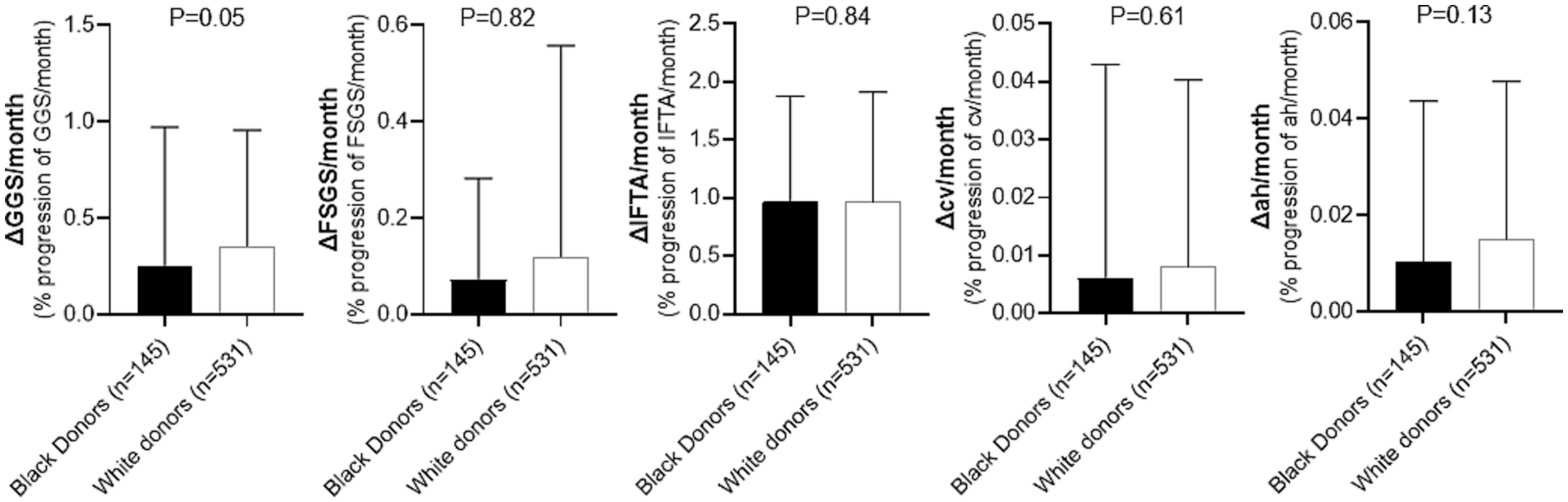
Comparison of histologic changes over time during follow-up period in recipients of kidney allografts from Black vs. White donors who did not develop CG. GGS, global glomerulosclerosis; FSGS, segmental glomerulosclerosis; IFTA, interstitial fibrosis/tubular atrophy; cv, arterial fibrointimal sclerosis; ah, arteriolar hyalinosis. Detailed comparison is presented in Supplementary Table S1.

Lastly, evaluation of the rate of change of histologic parameters over time for patients who did not develop CG revealed no statistically significant differences in any of the measured parameters when comparing recipients of kidney allografts from Black and White donors (Figure 4). Only a trend toward a lower rate of progression of GGS in recipients of kidneys from Black donors was observed (ΔGGS = 0.23 for Black donors vs. 0.27 for White donors, *p* = 0.05; Supplementary Table S1).

### CG and allograft failure

**Figure 5:**
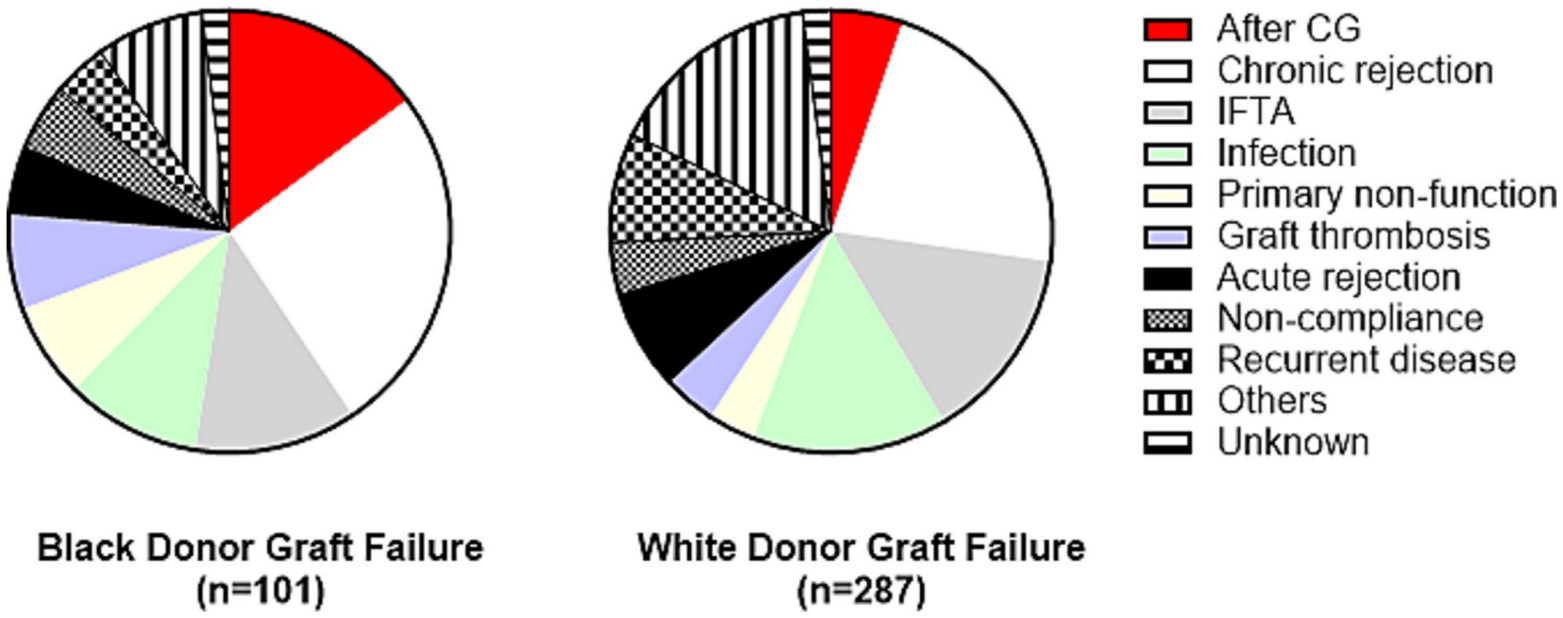
Causes of allograft failure in recipients of kidney allografts from Black vs. White donors. Only allograft failure after CG was significantly different between these two subgroups [OR = 3.16, *p* = 0.004]. Detailed comparison is presented in Supplementary Table S2. CG, collapsing glomerulopathy; IFTA, interstitial fibrosis/tubular atrophy.

When causes of allograft failure in recipients of kidney allografts from Black and White donors were compared, only allograft failure following the development of CG was more common in recipients of allografts from Black donors [*n* = 15 (15%) Black donors vs. *n* = 15, (5%) White donors; OR = 3.16, *p* = 0.004; Figure 5], while other causes were similar (Supplementary Table S2).

To explore the association of receiving kidneys from Black donors with allograft survival in patients who did not develop CG, time-to-event analyses were repeated after excluding the 58 allografts that developed CG during follow-up (30 from Black donors and 28 from White donors; Table 3). Notably, receiving a kidney from a Black donor was no longer associated with allograft failure (aHR = 1.24, *p =* 0.10), while receiving a kidney from a deceased donor (aHR = 2.02, *p* < 0.001) and presence of pre-transplant DSA (aHR = 1.45, *p* = 0.007) remained independently associated with allograft failure (Table 3).

**Table 3:** Univariable and multivariable analyses of death-censored allograft survival after excluding patients who developed CG.

Variables	Univariable (*n* = 1843)			Multivariable (*n* = 1814), N events = 353	
	N events	HR (95% CI)	*p* value	aHR (95% CI)	*p* value
Recipient age at transplant (per each year)	358	1.00 (0.99–1.01)	0.59	0.99 (0.99–1.00)	0.16
Recipient female gender	358	0.94 (0.76–1.16)	0.54	0.87 (0.70–1.08)	0.20
Recipient Black race	358	1.37 (1.09–1.73)	**0.008**	1.08 (0.84–1.39)	0.56
Donor age at transplant (per each year)^1^	357	1.00 (0.99–1.01)	0.64	1.01 (1.00–1.02)	0.05
Donor female sex	358	0.99 (0.80–1.22)	0.91		
Donor Black race	358	1.37 (1.07–1.74)	**0.01**	1.24 (0.96–1.61)	0.10
Allograft from Deceased donor	358	2.13 (1.70–2.66)	**<0.001**	2.02 (1.56–2.60)	**<0.001**
Previous transplant^2^	358	1.52 (1.18–1.96)	**0.001**	1.30 (0.98–1.73)	0.07
Pre-transplant DSA^3^	358	1.66 (1.31–2.10)	**<0.001**	1.45 (1.11–1.89)	**0.007**
# HLA mismatches (per antigen: 0–6)^4^	358	1.13 (1.05–1.21)	**0.001**	1.04 (0.96–1.12)	0.37
Induction with Depleting therapy	358	1.21 (0.91–1.60)	0.19		
Native kidney failure due to DM	358	1.05 (0.83–1.35)	0.68		
Native kidney failure due to FSGS	358	1.47 (1.06–2.07)	**0.02**	1.30 (0.92–1.83)	0.13
Median ZIP code household income (per each $10,000/year)^5^	353	0.96 (0.93–0.99)	**0.01**	1.00 (0.96–1.03)	0.81

DM, diabetes mellitus; DSA, donor-specific antibody; FSGS, focal segmental glomerulosclerosis.

1 Donor age: data on donor age were unavailable for 6 kidney donors (including 3 Black donors and 3 White donors).

2 Previous transplantation: data regarding previous renal transplantation were unavailable for 2 kidney recipients (both receiving kidneys from White donors).

3 Pre-transplant DSA: data regarding presence of pre-transplant DSA were unavailable for 1 kidney recipient (from White donor).

4 HLA-mismatch: data on number of HLA mismatches were unavailable for 1 kidney donor-recipient pair (with a White donor).

5 Median ZIP code household income: data regarding household income were unavailable for 21 kidney recipients (including 4 Black donors and 17 White donors).

Significant *P* values are in bold.

## Discussion

Most, although not all, studies have demonstrated shorter allograft longevity for recipients of kidney transplants from Black donors. Using data from the United Network for Organ Sharing (UNOS), Hariharan et al. found inferior allograft survival in recipients of kidney allografts procured from older deceased Black donors (1), while Callender et al. have expanded the above observations, and demonstrated that kidney allografts obtained from either deceased or living Black donors had shorter allograft survival (2). Using data from Veterans Affairs and US Renal Data System information, Taber et al. have also shown that recipients of kidney allografts from Black donors experience substantially reduced allograft survival (10). However, when restricting the evaluation to recipients of living donor transplants from the UNOS database, Isaacs et al. found that the effect of donor race was less demonstrable (11). Similarly, using UK Transplant Registry Data, Pisavadia et al. did not find a significant difference in allograft survival in recipients of kidney allografts from living or deceased Black donors when compared to those who received kidneys from White donors (12). Nevertheless, it should be noted that the aforementioned reports were registry-based studies performed on data from national kidney transplant tracking programs, where it is not possible to adjust for important confounders such as pre-transplant DSA, given the absence of these data, nor is it possible to study histopathologic changes in the allografts.

A single-center study found that Black donor race was associated with increased risk of allograft failure on multivariate analysis (HR = 1.56, *p* = 0.047) (13). However, that particular study was relatively small (including 118 kidneys from Black donors and 845 kidneys from White donors) and the investigators did not adjust for major immunologic confounders, including HLA-mismatch and the presence of pre-transplant DSA (13).

The KDPI is a tool that was developed in 2009 by Rao et al. with the intent of predicting the risk of allograft failure based on clinical and demographic characteristics of an eligible deceased donor (14). Ten donor characteristics, including Black vs. non-Black donor race, are used to calculate the KDPI (14). Unfortunately, the apparent negative impact of Black donor race on allograft survival and its inclusion in KDPI calculation have led to a higher discard rate for kidneys from Black donors (3), despite the fact that the pathophysiology behind such observations are still uncertain.

To understand the mechanisms underlying the observed racial disparities in kidney allograft survival, one must examine the histopathologic changes that occur in the kidney allograft. Development of CG in the kidney allograft is an infrequently encountered complication, but it is characterized by a dismal prognosis (4). While prior studies have suggested an association between CG and receiving kidney allografts from Black donors (4–6), none of these studies have compared the prevalence of CG between recipients of allograft kidneys from Black vs. White donors. Similarly, while accelerated vascular sclerosis and development of FSGS in native kidneys are more frequently observed in Black patients (15–18), a systematic assessment of the progression of such chronic changes in transplant recipients, stratified based on their donor races/ethnicities, is lacking.

Our study, which is the largest single-center investigation to date, and the first to assess histologic alterations in this patient population, has shown that, compared to recipients of kidneys from White donors, recipients of kidneys from Black donors incur only a modestly (19) increased risk of allograft loss (aHR = 1.34, *p* = 0.02), after adjusting for relevant risk factors, including recipients’ and donors’ demographics, kidney source, HLA-mismatches, history of previous transplantation, pre-transplant DSA, cause of native kidney failure, and socioeconomic status. Notably, recipients of kidney allografts from Black donors were more likely to be Black than those who received kidneys from White donors. The reasons for this are likely multifactorial, and include consanguinity (e.g., living-related donation), and attempts to match donor-recipient pairs with regard to blood group and HLA antigens, which are differentially distributed across different races/ethnicities (20, 21). Nonetheless, recipient race did not show an independent association with allograft survival after adjusting for other variables, including donor race.

With regard to histologic changes and outcome, the development of CG was more frequent in recipients of allograft kidneys taken from Black donors. Furthermore, when we examined the etiologies of allograft loss between kidneys from Black vs. White donors, the development of CG appeared to account for the majority of this observed disparity. In fact, patients who did not develop CG during follow-up showed similar rate of progression of other histopathologic parameters over time (including glomerulosclerosis, IFTA, and vascular sclerosis) in recipients of kidney allografts from Black and White donors. Moreover, receiving kidney allografts from Black donors lost its independent association with allograft failure after excluding patients who did not develop CG. Together, these findings support that CG is the main driver behind the worse prognosis observed in patients receiving kidneys from Black donors when compared to patients receiving kidneys from White donors.

Importantly, a few prior studies have shown that a relatively high proportion of patients who develop posttransplant CG have received allografts from donors with high-risk *APOL1* genotypes, which are increased in population with recent African ancestry (22). Moreover, a multicenter study demonstrated that the presence of high-risk *APOL1* genotypes was more appropriate indicator of risk of allograft failure than donor Black race (23). Therefore, it is plausible that the increased risk of allograft loss observed in kidneys procured from Black donors might be mediated by CG that develop following a second hit in patients, who received kidneys from donors with susceptible genetic background. Hence, precise identification of risk factors and molecular signals predisposing for the development of CG may increase utilization of kidney allografts from Black donors by identifying the minority of donors at the highest risk for CG. This might help eliminating a portion of the currently observed racial disparity in allograft survival, especially now that drugs are being developed to mitigate the effects of *APOL1* kidney-risk variants (24).

Some limitations of this report include the retrospective nature of the study and relying on self-defined race/ethnicity rather than genetic ancestry. The fact that this is a single-center study may also introduce recruitment bias. Furthermore, it is worth noting that data on donors’ race/ethnicity may also be difficult to obtain in some countries. Nevertheless, to our knowledge, this report is the first large study to compare the histologic changes in recipients of kidney allografts from Black vs. White donors with a special focus on CG. Another major strength of this report is the ability to account for different confounding factors.

Overall, our data show that recipients of allografts from Black kidney donors demonstrate modestly shorter allograft survival than recipients of White donor kidneys, and that this difference is driven by the development of CG in a minority of the allografts taken from Black donors, which negatively impacts allograft survival.

## Data availability statement

The original contributions presented in the study are included in the article/Supplementary materials, further inquiries can be directed to the corresponding author.

## Ethics statement

The studies involving humans were approved by IRB Columbia University Irving Medical center. The studies were conducted in accordance with the local legislation and institutional requirements. The ethics committee/institutional review board waived the requirement of written informed consent for participation from the participants or the participants’ legal guardians/next of kin because retrospective pathologic study where contacting the patients may not be possible.

## Author contributions

LD: Formal analysis, Writing – original draft, Data curation, Conceptualization. ED: Data curation, Writing – review & editing. GS: Writing – review & editing, Data curation. ST: Writing – review & editing, Data curation. DS: Writing – review & editing, Data curation. LR: Investigation, Writing – review & editing. VD’A: Conceptualization, Writing – review & editing. E-RV: Data curation, Writing – review & editing. SH: Formal analysis, Investigation, Writing – review & editing, Data curation. IB: Supervision, Formal analysis, Conceptualization, Writing – review & editing.
