# Supplementary material for: Collapsing glomerulopathy is likely a major contributing factor for worse allograft survival in patients receiving kidney transplants from black donors
Source: Front Med (Lausanne). 2024 Mar 14;11:1369225. doi: 10.3389/fmed.2024.1369225 (PMC10972956; doi:10.3389/fmed.2024.1369225)
Supplement: Supplementary file 1 [file Table_1.DOCX]

Supplementary Material

**Supplemental Table 1. Comparison of histologic changes over time during follow-up in** **recipients of kidney allografts from Black vs. White donors who did not develop CG.**

| **Histologic Parameters** | **Recipients of Kidney from Black Donors**  (n = 145) | **Recipients of Kidney from White Donors**  (n = 531) | ***P* – value** |
| --- | --- | --- | --- |
| ΔGGS (%) /month^1^ | 0.23 (0, 0.45) | 0.27 (0.02, 0.60) | 0.05 |
| ΔFSGS (%) /month^2^ | 0 (0.0, 0.09) | 0 (0.0, 0.12) | 0.82 |
| ΔIFTA (%) /month | 0.66 (0.38, 1.18) | 0.66 (0.35, 1.23) | 0.84 |
| Δcv (1-3) / month^3^ | 0 (0, 0.02) | 0 (0, 0.02) | 0.61 |
| Δah (1-3) / month | 0.01 (0, 0.02) | 0.01 (0, 0.03) | 0.13 |

Abbreviations: GGS, global glomerulosclerosis; FSGS, segmental glomerulosclerosis; IFTA, interstitial fibrosis/tubular atrophy; cv, arterial fibrointimal sclerosis; ah, arteriolar hyalinosis

^1^ Data on ΔGGS/month were unavailable for 1 patient (1 recipient of an allograft from a Black donor)

^2^ Data on ΔFSGS/month were unavailable for 1 patient (1 recipient of an allograft from a Black donor)

^3^ Data on Δcv/month were unavailable for 29 patients (4 recipients of allografts from Black donors and 25 recipients of allografts from White donors)

**Supplemental Table 2.** **Comparison of causes of graft failure in** **recipients of kidney allografts from Black vs. White donors.**

| **Causes of graft failure** | **Recipients of Kidney from Black Donors (n = 101)** | **Recipients of Kidney from White Donors (n = 287)** | ***P* – value** |
| --- | --- | --- | --- |
| **After collapsing glomerulopathy** | 15/101 (15%) | 15/287 (5%) | **0.004** |
| **Chronic rejection** | 26/101 (25%) | 63/287 (22%) | 0.49 |
| **IFTA** | 12/101 (12%) | 41/287 (14%) | 0.62 |
| **Infection** | 10/101 (10%) | 41/287 (14%) | 0.31 |
| **Primary non-function** | 7/101 (7%) | 10/287 (3%) | 0.16 |
| **Graft thrombosis** | 7/101 (7%) | 11/287 (4%) | 0.27 |
| **Acute rejection** | 5/101 (5%) | 21/287 (8%) | 0.49 |
| **Non-compliance** | 5/101 (5%) | 11/287 (4%) | 0.57 |
| **Recurrent disease** | 4/101 (4%) | 23/287 (8%) | 0.25 |
| **Others** | 8/101 (8%) | 45/287 (16%) | 0.06 |
| **Unknown** | 2/101 (2%) | 6/287 (2%) | 1.0 |

IFTA, interstitial fibrosis/tubular atrophy not otherwise specified.
